# Increased survival of experimentally evolved antimicrobial peptide-resistant *Staphylococcus aureus* in an animal host

**DOI:** 10.1111/eva.12184

**Published:** 2014-07-02

**Authors:** Adam J Dobson, Joanne Purves, Jens Rolff

**Affiliations:** 1 Animal & Plant Sciences, University of Sheffield, Western BankSheffield, UK; 2 Department of Genetics, Evolution and Environment, Institute of Healthy Ageing, University College LondonLondon, UK; 3 School of Life Sciences, Centre for Biomolecular Science, University of NottinghamNottingham, UK; 4 Institute of Biology, Free University BerlinBerlin, Germany; 5 Berlin-Brandenburg Institute of Advanced Biodiversity Research (BBIB)Berlin, Germany

**Keywords:** experimental evolution, immunity, pathogenecity, resistance evolution.

## Abstract

Antimicrobial peptides (AMPs) have been proposed as new class of antimicrobial drugs, following the increasing prevalence of bacteria resistant to antibiotics. Synthetic AMPs are functional analogues of highly evolutionarily conserved immune effectors in animals and plants, produced in response to microbial infection. Therefore, the proposed therapeutic use of AMPs bears the risk of ‘arming the enemy’: bacteria that evolve resistance to AMPs may be cross-resistant to immune effectors (AMPs) in their hosts. We used a panel of populations of *Staphylococcus aureus* that were experimentally selected for resistance to a suite of individual AMPs and antibiotics to investigate the ‘arming the enemy’ hypothesis. We tested whether the selected strains showed higher survival in an insect model (*Tenebrio molitor)* and cross-resistance against other antimicrobials *in vitro*. A population selected for resistance to the antimicrobial peptide iseganan showed increased *in vivo* survival, but was not more virulent. We suggest that increased survival of AMP-resistant bacteria almost certainly poses problems to immune-compromised hosts.

## Introduction

Antimicrobial peptides (AMPs) are major components of immune defences in multicellular organisms (Tzou et al. 2002; Koprivnjak and Peschel 2011). Bacterial resistance to AMPs is rarely observed in environmental or clinical isolates, leading some to suggest that these molecules are ‘resistance proof’ (Zasloff 2002). Based on these observations and the decline in effectiveness of antibiotics, AMPs have been proposed as the basis of synthetic drugs that could be used to fight human infection (Zasloff 2002; Reddy et al. 2004; Giuliani et al. 2007).

*In vitro* experiments have now demonstrated that, contrary to earlier expectations, AMPs are not resistance proof. Resistance evolved at low cost within just a few hundred generations in *Escherichia coli, Pseudomonas fuorescens* (Perron et al. 2006), *Salmonella enterica* (Pränting et al. 2008), *Streptococcus pneumoniae* (Habets et al. 2012) and *Staphylococcus aureus* (Dobson et al. 2013). Recent work has shown that pexiganan-resistant *S. aureus* can also be cross-resistant to human neutrophil defensin-1, a human AMP (Habets and Brockhurst 2012).

Importantly, the repertoire of AMP resistance mechanisms is limited (reviewed in Peschel and Sahl 2006; Koprivnjak and Peschel 2011; Gruenheid and Le Moual 2012). The fact that some microbes can utilise these few resistance mechanisms to inhabit AMP-rich environments, for example *Pseudomonas aeruginosa* in mucosal tissue (Gruenheid and Le Moual 2012), suggests that such mechanisms could be effective in conferring resistance to a broad range of AMPs. Thus, should therapeutic use of AMPs becomes a reality, providing selective conditions for the rise and spread of bacteria resistant to a broad spectrum of AMPs (cross-resistance), we risk ‘arming the enemy’ by equipping pathogens with tools for increased resistance to the immune system (Bell and Gouyon 2003; Buckling and Brockhurst 2005). To date, there has been no thorough test of the survival and virulence of a range of AMP-resistant bacteria in an animal host.

We recently demonstrated that fitness costs of AMP resistance are unlikely to constrain evolution in the gram-positive bacterium *S. aureus*. However, simultaneous selection at the same intensity by a combination of two AMPs constrained resistance (Dobson et al. 2013). The experimentally evolved strains from this study provide a resource with which we can comparatively explore the potential limits of shifts in survival of both bacteria and host after infection. AMPs play a critical role in insect innate immune defences (Moon et al. 1994; Lemaitre and Hoffmann 2007; Chae et al. 2012), which are therefore well suited to investigate the potential problems of AMP cross-resistance. As a model host, we use the mealworm *Tenebrio molitor*. This beetle exhibits a long-lasting humoral antimicrobial immune response to persistent *S. aureus* infection (Haine et al. 2008a), which is largely dependent on induction of AMPs (Johnston et al. 2014). We therefore predicted that infection titters in this system are sensitive to variation in AMP resistance. We used this host to parameterize survival of AMP-resistant bacteria *in vivo*, which we complemented with *in vitro* assays of cross-resistance. We also assessed the effects of these populations on survival of infected hosts. This allowed us to address the potential risk of AMP therapy for the first time in an animal host.

## Materials & methods

### Bacteria and stressor selection

We designed a proof-of-principle study to test the effects of AMP resistance on host and pathogen. All bacterial strains were *S. aureus* that we had previously selected for resistance against antibiotics or antimicrobial peptides (Dobson et al. 2013; Table 1). Briefly, these cultures were grown from an isogenic ancestor in the absence of selection for 10 days by serial passage. They were then inoculated into media containing one of the AMPs pexiganan, iseganan or melittin. Procedural controls were the antibiotics vancomycin or streptomycin. Untreated controls were inoculated into unsupplemented media. All AMPs/antibiotics were suspended at a concentration sufficient to inhibit 50% growth of the ancestral bacteria. Five replicate populations were established per AMP/antibiotic, and cultures were grown by daily serial passage. Concentration of each respective AMP/antibiotic was doubled weekly. Populations were grown for up to 4 weeks, or until extinction shortly after the beginning of week 3 (vancomycin treatment only). The ancestral population was constitutively tetracycline resistant, allowing us to selectively recover them from *T. molitor* postinfection on media containing tetracycline. These populations demonstrated equivalent performance in fitness assays.

**Table 1:** Bacterial populations pooled for *Tenebrio molitor* infection

Culture (treatment/timepoint)	Description [see Dobson et al. (2013) for protocol]
Ancestor	Preselection control. All other cultures were derived from this population
Iseganan, day 28	AMP-selected population. Selected by iseganan *in vitro* for 28 days. Highly resistant by *in vitro* assay.
Melittin, day 28	AMP-selected population. Selected by melittin *in vitro* for 28 days. Constituent populations showed variable resistance by *in vitro* assay.
Pexiganan, day 28	AMP-selected population. Selected by pexiganan *in vitro* for 28 days. Grew at low density for latter 14 days of selection. Re-assaying (this paper) demonstrates moderately increased resistance.
Unselected, day 28	Unselected day 28 control for drift in the absence of selection. Serially passaged in growth medium without stressors for 28 days.
Streptomycin, day 28	Antibiotic-selected control. Selected by streptomycin *in vitro* for 28 days.
Vancomycin, day 14	Antibiotic-selected control. Selected *in vitro* by vancomycin for 14 days. Showed apparent increase in resistance then rapid extinction on days 17–18.

### Bacteria preparation for infection and cross-resistance studies

Before infecting *T. molitor*, we pooled evolved populations from within treatments in equal proportion, for two reasons. First, ‘public goods’, in which one or a few clones in a bacterial population evolve traits that benefit the whole population, are known to mediate antibiotic resistance (Lee et al. 2010). AMPs can be inactivated by extracellular peptidases (Peschel and Sahl 2006; Koprivnjak and Peschel 2011), so population-level resistance, mediated, for example, by ‘public goods’, seems particularly likely for AMP-resistant bacteria. Second, real infections are commonly caused by mixed populations, not isogenic strains, and this has also been shown in *S. aureus* (Balmer and Tanner 2011). We therefore sought to assay the population-level processes relevant to a real infection, which can establish the limits of phenotypic space of AMP-resistant bacteria equally well as equivalent experiments with isolated clones.

Populations were pooled by partially thawing glycerol stocks until 100 μL could be removed, which was inoculated into 5 mL LB and grown to stationary phase. 500 μL aliquots of these cultures were spun for 5 min at 4500 g. Pellets from each treatment were then pooled by communal resuspension in 1 mL tryptic soy broth (Sigma-Aldrich T8907, Munich, Germany) with glycerol (80% water: 20% glycerol v/v) and frozen at −90°C.

Cultures of these pooled bacteria were prepared by directly inoculating 5 mL LB with a scrape of the pooled stock and grown for 24 h at 30°C with shaking. Twenty-four hour, cultures were pelleted by centrifugation at 4500 g for 5 min, then washed and resuspended in an equal volume of sterile PBS (NaCl 150 mm, Na_2_HPO_4_ 10 mm, pH 6.5).

To check for contamination and to quantify CFU in the inocula, 50 μL of each inoculated culture (diluted × 10^−5^) was plated with 20 sterile glass beads on LB (Sigma-Aldrich L2897) 1.5% agar containing tetracycline and amphotericin-B as previously. Plates were incubated for 24 h at 37°C and photographed. CFUs were automatically counted using OpenCFU (Geissmann 2013).

### *Tenebrio molitor* culture and infection

*Tenebrio molitor* were purchased as final-instar larvae from a commercial supplier (www.livefoods.co.uk) and grown in rat chow (Harlan Laboratories, Shardlow, UK) in an insectary at 26°C ± 1 in a 12:12-h light/dark cycle. F_1_ offspring of these beetles were used for infection experiments. Females were sexed as pupae and kept individually in rat chow, eclosion was recorded daily, and all beetles were virgins between 7 and 10 days old when used.

Pathogenicity and bacterial survival were assessed in *T. molitor* by injection with our experimentally evolved *S. aureus*. Beetles were surface-sterilized by swabbing the ventral abdomen with 80% EtOH, and loading a fine-pulled glass electrode (Narishige) with ∼10^5^ CFU inoculum suspended in 5 μL PBS. Inocula were injected between the third and forth abdominal sternites into the haemocoel. Negative controls were injected with 5 μL sterile PBS.

### Quantifying infection

*Staphylococcus aureus* were recovered from *T. molitor* 24 h after infection, coincident with peak AMP expression (Johnston et al. 2014). Controls were injected with sterile PBS to identify any tetracycline-resistant contaminants.

Bacteria were recovered using a ‘perfusion bleed’ assay, refined from that of (Haine et al. 2008b). The beetle cuticle was surface-rinsed for ∼45–60 s in 80% EtOH, to remove cuticular contaminants. Genitalia were everted by gently squeezing the abdomen and swabbed with 80% EtOH, and a small incision was made with a scalpel. A 30-gauge needle was inserted through the plural membrane exposed between the abdomen and thorax on the side of the body laterally opposite to the genital incision and pushed into the abdominal haemocoel. 500 μL sterile PBS was pushed through the needle, washing haemolymph out of the abdomen and into a sterile collection tube via the genital incision. 50 μL of perfused haemolymph samples was plated with 20 sterile glass beads on LB 1.5% agar plates containing tetracycline and amphotericin-B to select the experimental strains from the background insect flora. Plates were incubated and colonies counted as previously. CFU counts were then extrapolated to estimates per beetle. If bacteria grew at uncountably high densities, the sample was censored and assigned the maximum number of CFUs that could be counted by our assay (13 000).

### Pathogenesis assays

Beetles were injected 8 days after eclosion (50 individuals per treatment) as in the bacterial survival assay, and mortality of each individual was checked at least every 2 days. Mortality was recorded when beetles ceased to respond to mechanical stimulation.

### Cross-resistance assays

Cultures for *in vitro* cross-resistance assays were grown from pooled stocks in Müller-Hinton broth (Sigma-Aldrich 70192, 5 μg mL^−1^ tetracycline, 5.6 μg mL^−1^ amphotericin-B) for 18 h at 30°C with shaking, to a density of 5 × 10^6^ CFU mL^−1^. We tested resistance of our evolved populations to the antimicrobial peptides pexiganan (courtesy of Michael Zasloff, Georgetown University, USA), melittin (Sigma-Aldrich M2272) and a 50:50 combination of the two (PGML). Unfortunately, we were unable to obtain sufficient iseganan for a fully reciprocal design. Resistance of naive bacteria to these antimicrobials (cross-resistance) was determined by dose-response assays in 100 μL volumes per well in sterile 96-well microtitre plates, using a twofold dilution series from 64 to 0.125 μg mL^−1^ and an additional well of unsupplemented Müller-Hinton broth. 10 μL of culture was added to each well and OD_595_ was measured every hour for 6 h, allowing for exponential growth. MIC was determined as the first concentration in which no growth was detected after 6 h.

### Data analysis

PBS-injected controls revealed no contamination and so were excluded from further analyses. We performed two analyses of CFU counts. All data were analysed in R version 3.0.2 (R Core Team 2013). To account for censored data from overgrown plates, we used the MCMCglmm package (Hadfield 2010). This innovative package uses Markov chain Monte Carlo techniques to fit generalized linear models in a Bayesian framework, with a wide range of potential error distributions. These distributions include the censored Poisson distribution, which we used to account for censored data from our CFU counts. This model was fitted with 1.3 million iterations, thinning interval of 1000 and a burn-in of 300 000 generating posterior coefficients for each treatment group with 95% confidence intervals. To verify these results, we additionally fitted a standard frequentist GLM with a quasi-Poisson distribution after excluding the censored samples, using the R base GLM function. *Post hoc* multiple comparisons of frequentist GLM were performed using the general linear hypothesis testing (glht) function from the R multcomp package. The two approaches yielded congruent results, so we present results from the more powerful MCMCglmm.

Survival of beetles in pathogenesis assays was censored 21 days after eclosion. The data were analysed with an accelerated failure time (AFT) survival model with a Weibull distribution, using the survival package (Therneau 2013).

## Results

### Selected *Staphylococcus aureus* resistance in *Tenebrio molitor*

**Figure 1:**
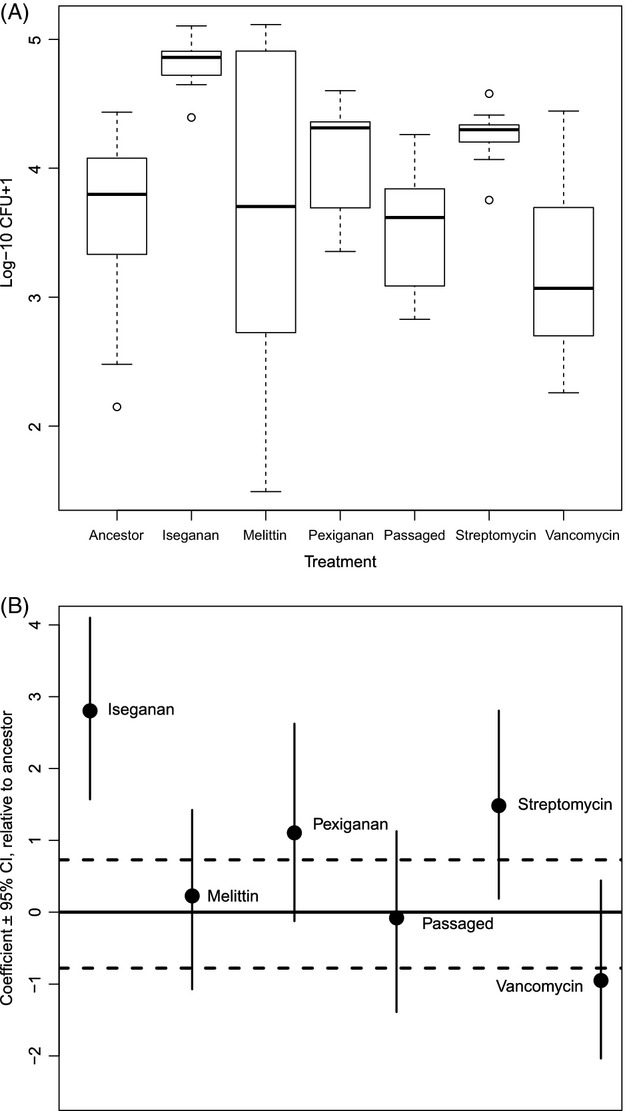
24-h *Staphylococcus aureus* persistence in *Tenebrio molitor*. AMP- and antibiotic-selected *S. aureus* populations were inoculated into *T. molitor*, and recoverable cells quantified after 24-h exposure. (A) The box and whisker plots present the density of each bacterial population in *T. molitor* as the average log^10^ CFU per beetle, showing medians, first and third quartiles and 5th and 95th percentiles. (B) MCMC techniques were used to fit a generalized linear model to the CFU data, because certain plates were uncountable and were therefore right-censored. The MCMCglmm used a censored Poisson distribution to estimate differences between treatments. Solid line at 0 represents the intercept (ancestral population), ±95% confidence intervals represented by dashed lines. The points represent coefficients of each treatment ±95% confidence intervals. Iseganan (p_MCMC_ = 0.002), streptomycin (p_MCMC_ = 0.02) and pexiganan (p_MCMC_ = 0.052) showed significantly more immunoresistance than the ancestor strain. Frequentist GLM and multiple comparisons are presented in supplementary material.

There was significant treatment-specific variation in survival of selected *S. aureus* populations 24 h after inoculation into *T. molitor* (Fig. 1A and B, Table 2). Of the AMP-selected lines, iseganan-selected bacteria showed higher survival in the host than the ancestor (p_MCMC_ = 0.002), and pexiganan-selected bacteria showed a trend towards higher survival (p_MCMC_ = 0.052). Melittin-selected bacteria did not survive significantly better than the ancestral population, but there was significantly more variance in this group than in the rest of the data set together (*F*-test, *F* = 2.48, df = 8, 64, *P* < 0.05), suggesting a more diverse mix of resistant and nonresistant clones in this inoculum than in those in other treatments. Relative to the ancestral population, vancomycin-selected and unselected lines showed no differences (p_MCMC_ > 0.1). Streptomycin-selected bacteria showed increased survival compared with the ancestral strain as well as the unselected control strain (p_MCMC_ = 0.02). Results from a frequentist GLM, along with Tukey's multiple comparisons, are presented in Table S1.1 and S1.2.

**Table 2:** Analysis of persistence of selected bacteria (CFU counts) *in vivo* by MCMCglmm with censored Poisson distribution

Treatment level	Posterior mean (coefficient)	−95% confidence interval	+95% confidence interval	p_MCMC_
Intercept (Ancestor)	5.98	5.29	6.72	<0.001
Iseganan	+2.81	+1.57	+4.16	0.002
Melittin	+0.28	−0.91	+1.55	0.632
Pexiganan	+1.12	−0.02	+2.40	0.052
Unselected	−0.12	−1.42	+1.06	0.832
Streptomycin	+1.49	+0.05	+2.61	0.020
Vancomycin	−0.94	−2.15	+0.31	0.134

### Pathogenicity of selected *Staphylococcus aureus* to *Tenebrio molitor*

**Figure 2:**
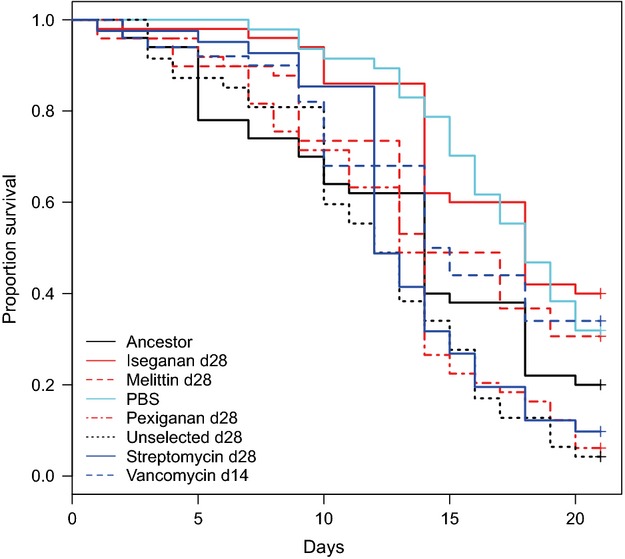
Postinfection survival of *Tenebrio molitor* infected with selected and unselected *Staphylococcus aureus*. The proportion of *T. molitor* survival after infection with each bacterial population and PBS control is presented over 21 days. Summary statistics are presented in Table 3.

Bacterial selection line had significant effects on the pathogenicity of some strains (Fig. 2). Our iseganan-selected bacteria became less pathogenic streptomycin- and pexiganan-selected and unselected controls (Table 3). Comparisons between beetles injected with PBS to beetles infected with bacteria revealed the same pattern of differences as seen in the iseganan group, indicating that treatment by iseganan reduced pathogenicity of the population. Pexiganan-selected and unselected bacteria were also more pathogenic than the vancomycin-selected population.

**Table 3:** Comparisons of survival *Tenebrio molitor* (parametric survival model with Weibull distribution) infected with AMP or antibiotic-selected *Staphylococcus aureus*. Significant effects are emboldened

Treatment A	Treatment B	Estimate	SE	Z	Adjusted P
Iseganan	Ancestor	0.2812	0.10148	2.771	0.10201
Melittin	Ancestor	0.12926	0.09758	1.325	0.8892
PBS	Ancestor	0.23816	0.09949	2.394	0.24299
Pexiganan	Ancestor	−0.14656	0.09035	−1.622	0.73596
Unselected	Ancestor	−0.19249	0.09083	−2.119	0.40125
Streptomycin	Ancestor	−0.10046	0.0953	−1.054	0.96572
Vancomycin	Ancestor	0.15832	0.09844	1.608	0.74472
Melittin	Iseganan	−0.15194	0.10483	−1.449	0.83313
PBS	Iseganan	−0.04305	0.1062	−0.405	0.99992
**Pexiganan**	**Iseganan**	−**0.42776**	**0.09885**	−**4.327**	**<0.001**
**Unselected**	**Iseganan**	−**0.47369**	**0.09939**	−**4.766**	**<0.001**
**Streptomycin**	**Iseganan**	−**0.38166**	**0.10313**	−**3.701**	**0.00537**
Vancomycin	Iseganan	−0.12288	0.1055	−1.165	0.9415
PBS	Melittin	0.10889	0.10299	1.057	0.96512
Pexiganan	Melittin	−0.27582	0.09473	−2.912	0.07005
**Unselected**	**Melittin**	−**0.32175**	**0.09523**	−**3.379**	**0.01647**
Streptomycin	Melittin	−0.22972	0.09934	−2.312	0.28519
Vancomycin	Melittin	0.02906	0.10211	0.285	0.99999
**Pexiganan**	**PBS**	−**0.38471**	**0.09677**	−**3.975**	**0.00164**
**Unselected**	**PBS**	−**0.43064**	**0.0973**	−**4.426**	**<0.001**
**Streptomycin**	**PBS**	−**0.33861**	**0.10119**	−**3.346**	**0.01842**
Vancomycin	PBS	−0.07984	0.1037	−0.77	0.99456
Unselected	Pexiganan	−0.04593	0.08761	−0.524	0.99954
Streptomycin	Pexiganan	0.0461	0.0923	0.499	0.99966
**Vancomycin**	**Pexiganan**	**0.30488**	**0.09564**	**3.188**	**0.03111**
Streptomycin	Unselected	0.09203	0.09279	0.992	0.97557
**Vancomycin**	**Unselected**	**0.35081**	**0.09615**	**3.648**	**0.00647**
Vancomycin	Streptomycin	0.25878	0.10018	2.583	0.16099

### Assessment of cross-resistance *in vitro*

**Figure 3:**
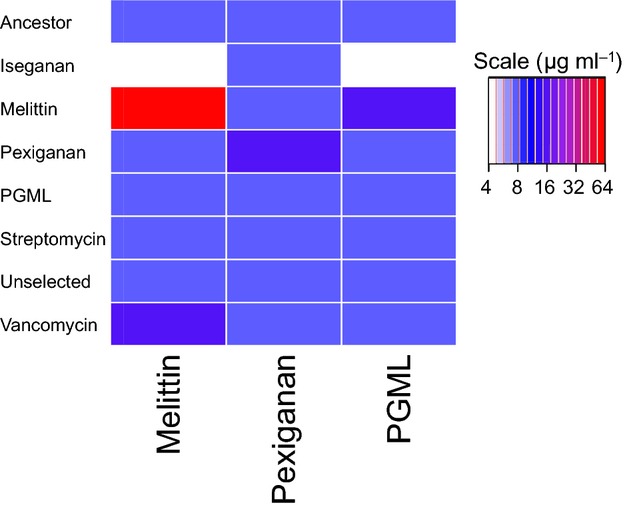
Cross-resistance of pooled selected cultures to antimicrobial peptides. AMP- and antibiotic-selected *Staphylococcus aureus* populations were assessed for resistance to pexiganan, melittin and PGML using a standard MIC dilution plate assay (*n* = 3). Intensity of resistance of a selection line to each stressor is indicated by colour, ranging from white (MIC = 4 μg mL^−1^) to red (MIC = 64 μg mL^−1^).

We did not find strong evidence of *S. aureus* cross-resistance to AMPs through *in vitro* assays (Fig. 3). Relative to the ancestor, melittin- and pexiganan-selected bacteria showed increased resistance to the compounds that they had been selected by. Melittin-selected bacteria additionally showed increased resistance to PGML. Iseganan-selected bacteria showed decreased resistance to melittin, pexiganan and PGML, suggesting a cost of iseganan resistance in this context. All other bacteria showed equal pexiganan, melittin and PGML susceptibility as the ancestor.

## Discussion

Our studies show increased survival of iseganan-resistant *S. aureus in vivo*, consistent with the hypothesis of AMP therapy having the potential to ‘arm the enemy’. This result associates evolved resistance to a single AMP, as would be the case in a topical application, with enhanced survival in the face of an AMP-dependent immune response. Our streptomycin-selected procedural control showed somewhat increased survival in the beetle. As streptomycin interferes with bacterial ribosomes – which to our knowledge are not affected by insect immune systems – without further functional studies, this result can only be attributed to increased ‘vigour’ after selection (Kawecki et al. 2012). Nevertheless, the iseganan-selected bacteria survived in the host on average 3.7 times better (adjusted *P* < 0.005) than the streptomycin-selected bacteria (Fig. 1, Table S1.2.), which can be most parsimoniously attributed to differences arising from selection for iseganan resistance. Further work is required to attribute these effects to specific mechanisms.

We observed some congruence between *in vivo* survival of bacteria in this study and *in vitro* resistance in our previous (Dobson et al. 2013), but this pattern must be interpreted with caution. Iseganan-selected bacteria were highly iseganan-resistant *in vitro* and also survived well in *T. molitor*. Melittin-selected bacteria showed striking interpopulation variability in resistance *in vitro*, and considerable variation in the numbers of bacteria recovered from beetles. However, our pexiganan-resistant bacteria, which performed poorly under selection *in vitro*, survived somewhat better *in vivo*. Additionally, the observed increase in survival in the host could not be clearly predicted from the *in vitro* cross-resistance data presented here. This highlights that *in vivo* testing is crucial to explore the potential of cross-resistance in a natural context (Martinez 2008). It would be of value in future to assess how many mutations arose in iseganan-selected strains, and whether genetic variants associated with improved survival in the host are also associated with iseganan resistance. As there are no data on the number of generations that each strain grew for under selection, we cannot estimate the number of mutations that arose in each of our populations, and whether these were equivalent across treatments. However, these strains were all able to grow to roughly equivalent density (optical density, 595 nm) in their respective growth media, and there were no differences between the fitness index (calculated from growth rate) of populations selected for resistance to iseganan or streptomycin (Dobson et al. 2013), indicating that effects in the iseganan-selected bacteria are not likely due to accrual of more mutations than other populations.

Increased *in vivo* survival of AMP-selected bacteria raises concerns about the eligibility of AMPs as therapeutic antimicrobial or immunomodulatory drugs (Hancock 2000; Zasloff 2002; Easton et al. 2009; Yeung et al. 2011). Our *in vivo* data augment the proposition that clinical use of AMPs could select widespread resistance to the immune system. Habets and Brockhurst (2012) demonstrated *in vitro* cross-resistance to humanneutrophil defensin-1 in pexiganan-resistant *S. aureus,* although this effect was not universal amongst resistant clones. However, Pränting et al. (2008) were unable to identify cross-resistance to a panel of AMPs and antibiotics in an isogenic *Salmonella enterica* sbaA mutant resistant to the porcine AMP PR-39 (Pränting et al. 2008). Collectively, these results and ours support the notion that cross-resistance and immunoresistance of AMP-resistant bacteria is context-dependent (Habets and Brockhurst 2012): it is possible, but not a universal feature of AMP-resistant clones. Also, in our study, the *in vitro* assay was less informative than our *in vivo* assay. This highlights the importance of using robust *in vivo* models to parameterize performance of resistant microbes. Further work is required to determine the genetic bases of broad-spectrum AMP resistance in these bacteria.

It was surprising that iseganan-selected bacteria showed higher survival *in vivo* but were less pathogenic than our unselected, streptomycin- and pexiganan-selected bacteria. Despite this disparity, any antimicrobial therapy that confers an advantage to infectious bacteria *in vivo* is clearly undesirable. One possibility is that the iseganan-selected bacteria have evolved mutations that facilitate better survival in the host mediated by reduced activation of the immune system (Atilano et al. 2011). An additional possibility is that bacteria at high-density negatively regulate virulence traits to maximize transmission (Antia et al. 1994) or that mortality is too crude a measure of pathology to quantify costs to the host of infection with AMP-resistant bacteria. Our design, using mixed populations rather than individual clones, does not allow us to make causal links between resistance and pathogenicity of the selected strains because individual clones within the populations may cause different phenotypes. It is beyond the scope of the present manuscript to determine the mechanistic basis of the reported phenotypes, but future characterization of isogenized strains from these populations could be leveraged to test of the role of AMPs in mediating trade-offs between resistance and pathogenicity. As subcutaneous *S. aureus* must putrefy its host for transmission, such a trade-off would indicate stabilizing selection on AMP resistance.

In medical settings, the processes described above may be exacerbated. AMPs are an important part of the repertoire of mammalian immune defences (Peschel and Sahl 2006; Kraus and Peschel 2008; Koprivnjak and Peschel 2011; Gruenheid and Le Moual 2012). Our finding, that bacteria that have evolved resistance to antimicrobial peptides can survive better in vivo, complements the finding of Habets and Brockhurst (2012) that pexiganan-resistant *S. aureus* can be cross-resistant *in vitro* to the human AMP HNP-1. The feasibility of AMPs as future antimicrobial therapies is therefore seriously compromised: resistant nosocomial infections could pose serious hazards, particularly for immune-compromised patients.

In summary, we have shown that an opportunistic pathogen can be fitter in a host after selection for antimicrobial peptide resistance. To our knowledge, this is the first study linking *in vitro* antimicrobial peptide resistance to *in vivo* survival. The therapeutic use of a single AMP against infection could ‘arm the enemy’ (Bell and Gouyon 2003) with cross-resistance to AMPs, including those produced as part of human immune responses.

## Data archiving statement

Data from this study are available from the Dryad Digital Repository: http://dx.doi.org/10.5061/dryad.f80bh.

## Supporting Information

**Table S1.** Table S1.1 Analysis of persistence of selected bacteria (CFU counts) *in vivo* by glm with quasipoisson distribution. Table S1.2 Multiple contrasts (Tukey) of persistence of selected bacteria.
