# Supplementary material for: Increased survival of experimentally evolved antimicrobial peptide-resistant Staphylococcus aureus in an animal host
Source: Evol Appl. 2014 Jul 2;7(8):905–12. doi: 10.1111/eva.12184 (PMC4211720; doi:10.1111/eva.12184)
Supplement: Supplementary file 1 [file eva0007-0905-sd1.docx]

| Table S1.1 Analysis of persistence of selected bacteria (CFU counts) *in vivo* by glm with quasipoisson distribution | | | | |
| --- | --- | --- | --- | --- |
| Treatment level | Coefficient | Standard Error | t value | P value |
| Intercept (Ancestor) | 8.97 | 0.04 | 51.99 | <2e-16 |
| Iseganan | +2.22 | 0.06 | 4.57 | 2.53e-07 |
| Melittin | +1.61 | 0.07 | 1.14 | 2.0e-4 |
| Pexiganan | +0.81 | 0.07 | 1.88 | 0.10 |
| Unselected | -0.28 | 0.07 | -0.13 | 0.66 |
| Streptomycin | +0.91 | 0.07 | 2.50 | 0.06 |
| Vancomycin | -0.51 | 0.07 | 1.70 | 0.47 |

| Table S1.2 Multiple contrasts (Tukey) of persistence of selected bacteria | | | | | |
| --- | --- | --- | --- | --- | --- |
| Treatment A | Treatment B | Estimate | Standard error | Z value | P value |
| iseganan | ancestor | 2.2256 | 0.3886 | 5.727 | < 0.001 |
| melittin | ancestor | 1.6115 | 0.4109 | 3.922 | 0.00145 |
| pexiganan | ancestor | 0.8099 | 0.4845 | 1.672 | 0.60819 |
| Unselected | ancestor | -0.279 | 0.6478 | -0.431 | 0.99943 |
| streptomycin | ancestor | 0.9191 | 0.4725 | 1.945 | 0.422 |
| vancomycin | ancestor | -0.51 | 0.7046 | -0.724 | 0.98979 |
| melittin | iseganan | -0.6141 | 0.2673 | -2.298 | 0.22356 |
| pexiganan | iseganan | -1.4157 | 0.3706 | -3.82 | 0.00224 |
| Unselected | iseganan | -2.5046 | 0.5677 | -4.412 | < 0.001 |
| streptomycin | iseganan | -1.3065 | 0.3548 | -3.682 | 0.00386 |
| vancomycin | iseganan | -2.7357 | 0.6318 | -4.33 | < 0.001 |
| pexiganan | melittin | -0.8016 | 0.3939 | -2.035 | 0.36478 |
| Unselected | melittin | -1.8905 | 0.5832 | -3.242 | 0.01779 |
| streptomycin | melittin | -0.6924 | 0.3791 | -1.826 | 0.50108 |
| vancomycin | melittin | -2.1215 | 0.6457 | -3.286 | 0.01549 |
| Unselected | pexiganan | -1.0889 | 0.6372 | -1.709 | 0.58255 |
| streptomycin | pexiganan | 0.1092 | 0.4578 | 0.238 | 0.99998 |
| vancomycin | pexiganan | -1.32 | 0.6949 | -1.9 | 0.45196 |
| streptomycin | Unselected | 1.1981 | 0.6282 | 1.907 | 0.44657 |
